# On the path to generate electricity from wastewater through genetic engineering of *Escherichia coli*

**DOI:** 10.1093/synbio/ysae002

**Published:** 2024-01-16

**Authors:** Charlotte Ayn Cialek

**Affiliations:** Inscripta Inc., Boulder, CO, USA

Developing sustainable production schemes by recycling industrial waste streams can avoid the pollution of traditional methods and reduce our dependency on fossil fuel. To achieve this, scientists have engineered microorganisms to convert waste, such as whey from the cheese industry, cellulose from agriculture, and wastewater from the brewing industry, into valuable products such as nutritional protein, nutraceuticals, and biofuels (1–3). But food and fuel are not the only products we can dream of making from waste: some microorganisms have the astounding natural ability to generate electricity from their metabolism. If scaled and customized, this capability could be harnessed to generate electricity from waste. However, natural electrogenic microorganisms are not easily cultivated on waste streams or genetically engineered in a laboratory setting.

To overcome this hurdle, a team led by researchers at the Swiss Federal Institute of Technology (EPFL) in Lousanne, Switzerland, successfully equipped *Escherichia coli* with the capacity to generate electricity and showed that *E. coli* could metabolize brewery wastewater (4). This new electrogenic *E. coli* strain brings us one step closer to achieving a waste-fed green-energy fuel cell.

Naturally electrogenic microorganisms, such as *Shewanella oneidensis*, can generate electricity by converting the energy gained from their food into an electron flow. This process is called extracellular electron transfer (EET). Since electrons do not naturally flow out of cells, *S. oneidensis* accomplishes EET using an assortment of chaperone proteins to transport electrons across membranes. Devices can capture this directional electron flow using an electrode as final electron acceptor.

Unlike *S. oneidensis*, the well-studied microorganism *E. coli* is not naturally electrogenic. Previous work achieved EET in *E. coli* by adding genes from naturally electrogenic microorganisms (5–10). However, the engineered *E. coli*’s electrogenic capabilities have, so far, remained low efficiency and poorly understood. To solve this, the research team at EPFL installed in *E. coli* various combinations of genes, including its natural cytochromes and EET-pathway proteins from electrogenic microorganisms. Through testing these variants, they not only discovered an optimized combination of EET genes but also identified electron transfer bottlenecks in the pathway.

A hurdle to efficient EET in *E. coli* is transferring electrons across its not one but two outer membrane layers. The periplasm is a 20–30 nm wide space between the internal and external membranes of *E. coli*. Thus, shuttling electrons out of the cell requires multiple transportation steps. Additionally, efficient EET requires equivalent rates of the transportation steps, such that the flux into the periplasm matches the flux out of the cell. The authors tested different combinations of genes from *S. oneidensis* and *E. coli* with the metal reducing extracellular electron transfer (Mtr) pathway from *S. oneidensis.* Ultimately, they found a combination of heterologous genes that, when expressed in *E. coli*, could act as an assembly line to pass electrons from the internal cellular space to the periplasm, then across the external membrane and outside of the cell. By using colorimetric assays and voltage measurements, the researchers could compare combinations of EET-pathway protein variants and cofactors to achieve an optimized electron flow.

Achieving improved EET in the versatile, engineerable microorganism *E. coli* can provide a platform for green electricity generation. As shown in this work, unlike natural electrogenic microorganisms, *E. coli* is capable of metabolizing brewery wastewater. Thus, EET-capable *E. coli* could potentially convert brewery wastewater into electricity, which could be harvested by charging a fuel cell. Since *E. coli* can metabolize other types of waste streams, brewery waste processing only scratches the surface of the green capabilities of *E. coli*-generated bioelectricity. Beyond waste processing, EET can be utilized for a variety of applications, including biomining, bioremediation, and biocatalysis.

Though this work provides an improvement on EET-capable *E. coli*, reaching sustainable bioelectricity generation will likely require increased rate and efficiency of EET and substrate metabolism. These processes can likely be tackled by genetically engineering *E. coli*’s well-studied, well-mapped metabolism (11). Engineering these capabilities may result in a microorganism that can not only tolerate but thrive in waste substrates.
